# Lectures on the Diagnosis and Treatment of Diseases of the Heart

**Published:** 1841-09-25

**Authors:** W. W. Gerhard


### MEDICAL EXAMINER.

DEVOTED TO MEDICINE, SURGERY, AND THE COLLATERAL SCIENCES.

No. 39.] PHILADELPHIA, SATURDAY, SEPTEMBER 25, 1841. [Vol. IV.

### LECTURES ON THE DIAGNOSIS AND TREATMENT OF DISEASES OF THE HEART.

BY W. W. GERHARD, M. D.

### LECTURE XVI.

#### DISEASES OF THE HEART.

The signs of the diseases of the heart are more easy, but less precise than those of the lungs. The structure of the heart is extremely simple, and its functions are very limited; while each of its surfaces is covered by a serous membrane which is the subject of much fewer lesions than the complex tissue of the lungs. The little complexity of the structure of the heart has its disadvantages for diagnosis; there is no expectoration from a mucous surface, and not the numerous combinations of rhonchi met with in the diseases of the lungs, which, although sometimes difficult to recognise, are generally sufficient to point out, with great accuracy, the exact nature and seat of the lesion. So far as the signs of diseases of the heart go, they are, therefore, very easy of recognition; but, beyond a certain point, they do not indicate the nature of the lesion with much precision, and the diagnosis is then approximative only. The gradual researches of late pathologists have, however, removed much of this difficulty, and although we have not yet reached precision, it is more nearly attained than it formerly was; and many disorders, such as inflammation of the lining membrane, and some valvular diseases, are much more easily recognised than they formerly were. This accuracy in diagnosis will probably extend a little further, although we doubt whether it will attain absolute perfection, so as to enable us to recognise the slighter organic lesions; this, however, is not, in most cases, of great practical importance.

The discovery of auscultation has probably done still more for the study of the diseases of the heart than of the lungs; that is, they were almost totally unknown, except the description of some of the pathological lesions. The symptoms of the different affections are so nearly allied, and so often obscured by those of various disorders of the lungs that it was extremely difficult to distinguish them one from the other, or even in many cases to decide that any affection of the heart existed. The united influence of accurate observation aided by physical exploration, and of pathological anatomy, has removed these difficulties as much as the nature of the subject will admit; and, as a necessary result, the progress of investigation has been directed to the causes which precede cardiac affections, and to the numerous secondary disorders which result from them. Hence, the disease of the heart is taken as a starting point; and the secondary affections are either lost sight of, or are properly regarded as mere effects, not as separate disorders. Thus, the congestions of the lungs, and the serous effusions into the chest, are, comparatively speaking, rarely mentioned; and hydrothorax, and asthma—when it is a mere consequence of heart disease—now attract little attention.

The investigation as to the causes of heart disease has produced some unlooked for results, and has shown very conclusively that in a large majority of cases, especially in young persons, they result directly from inflammation; and that even in the aged, inflammation is a secondary cause which adds very much to the slow alterations of nutrition, which arise merely from advance in years. As the causes are now better known, the treatment of these affections has become more definite,—that is, the early treatment employed with perseverance during the early or inflammatory period, before those fixed organic lesions are formed which are beyond the reach of art.

After this period our resources are more limited, and are strictly palliative, so far as the cardiac lesion itself is concerned, and our object is then rather to prevent the increase of the lesion and to relieve its effects upon other organs, than to remove it. We regard organic alterations when fixed, and, as it were, established, very nearly in the same light as original vices of conformation, from which they differ very little. The curative treatment is then applicable only to the reactive inflammatory stage, or to the early periods of the disease, in case it is not inflammatory at its commencement. Treatment may then be active and positive in its results.

Although in those stages in which the organic lesion is fixed and has become a mere peculiarity of nutrition, we cannot directly remove it, the mere prevention of increase often allows the natural powers of the system to recover the balance to which they are perpetually tending. In this way a considerable enlargement of the heart will sometimes gradually diminish, until the organ is gradually restored to its natural dimensions. These cures, however, must be limited to those cases in which the diseased part is enlarged, and the new superfluous portion of structure may then be absorbed; but when there is a destruction of an important part, or an entire perversion of its tissue, a cure can in no case be expected, and the treatment is then absolutely palliative.

*Symptoms of Diseases of the Heart.*—These are to some extent common to all those affections, whether functional or organic, but they vary extremely in intensity, and are by no means directly proportioned to the severity or the danger of the affection. The principal symptoms which occur in most diseases of the heart are irregular and disordered action of the organ, sometimes amounting to that degree of violence which is commonly called palpitation; painful or disagreeable sensations in the region of the heart; and impediments to the circulation, causing congestions of blood, and effusions of serum. Palpitation of the heart is more constant and troublesome to the patient in simple nervous disorder than in organic disease; in the latter case it is usually provoked only by violent exercise, or by some sudden effort. In the acute inflammatory cases the symptom is often totally absent. Painful sensations in the chest are very variable, one of the most distressing is an acute pain felt near the left nipple, or at the extremity of the sternum; this pain, it is true, does not always coincide with any positive symptoms of cardiac disease, but, in many cases, is plainly connected with a mere nervous disorder, or with dilatation, or with both these conditions combined. The pain is not accompanied with dyspnœa, as in angina pectoris, but it will sometimes extend across the chest or pass down the left arm. Both palpitations and pain are as often connected with nervous disease as with organic lesion; but this is not the case with the impediments to the circulation and their effects; these are almost always dependant upon organic disease, or, at least, much more frequently, and to a much greater degree than upon organic lesion. They occur in muscular derangements of structure, as hypertrophy and dilatation, but are much more decided if the valves are at the same time diseased. As a general rule they are more severe in proportion as the valves are narrowed, so as to prevent the free passage of the blood, forcing it, as it were, backwards, and thus producing congestions and anasarcous effusion, or hydrothorax. When the symptoms of heart disease have for a long time preceded the dropsy, they may be regarded as almost pathognomonic of a grave lesion, which is in these cases most frequently hypertrophy conjoined with valvular disease.

Irregularity and intermittence of the pulse attracted more notice before the discovery of auscultation than it does at present; for although this symptom is not without its value, and in reality often attends various heart diseases, it is necessarily uncertain, and sometimes occurs during the convalescence of acute diseases in which the heart is in no wise involved, while it is a congenital peculiarity in some individuals, lasting through a long life, but apt to terminate in decided heart disease. It is clearly not owing directly to the obstruction, but to the enfeebled action of the heart, which is no longer proportioned to the column of blood which it has to propel, and works in a hesitating irregular manner. Now this may arise from causes totally independent of actual disease of the heart, but it is more apt to occur in connection with heart disease than independently of it; and in other cases where no actual disorder is developed, the chances of future affections of the heart are certainly increased.

*Causes of Heart disease.*—The inflammations of the membranes of the heart not only constitute a frequent form of disorder, but give rise to a large proportion of organic lesions; this is more especially the case with the inflammation of the internal membrane, for pericarditis has comparatively little influence in producing permanent derangement of structure. The causes of these inflammations resolve themselves in those which ordinarily produce the phlegmasia, and into the peculiar connection known to exist between them and rheumatic disease. Besides inflammation, there are, however, other causes of heart disease; the muscular tissue of the organ may increase in thickness from the constant activity into which it is thrown, and organic disease is in this way developed as a consequence of long continued nervous excitement. Enlargement of the heart may also arise from a sudden injury inflicted upon it as a violent strain or effort, or some other sudden propulsion of the blood towards the organ which is strained beyond the power of complete recovery. The gradual advance of age has also a tendency to produce a gradual enlargement of the heart and the formation of ossific deposits in the valves or its internal membranes; and in these cases there is at least no evidence of direct inflammatory action. The causes of functional diseases of the heart are, of course, as various as of all nervous disorders, and are sometimes the most opposite in their character; in general, the nervous disorders are apt to arise in cases of anemia, or of deficient muscular power, or are directly dependent upon spinal irritation or chronic gastric disorder.

*Terminations of Heart disease.*—The acute inflammatory affections of the heart may terminate in recovery, and the patient may be restored to entire health; but in many cases the disease gets well so far as the acute affection is concerned, but the organic lesion continues. Chronic organic affections, as a general rule, do not terminate in recovery; they may end in death, or they may be prolonged without causing more than mere discomfort to the patient, or shortening the natural duration of his life. The former case arises from the severity of the lesion, which is often sufficient to seriously impede the circulation of the blood; or from the enfeebled state of the patient, and the thinness of the blood, which favours the dropsical effusions of the latter stages of these diseases. These aggravated cases vary in duration, but they generally prove fatal of themselves, or they merely increase the severity and the danger of some intercurrent disease, so that death results from the combined influence of the chronic and of the acute disease. The structure and peculiar functions of the heart increase the mortality from the chronic diseases; they are rarely single, or, at least, do not long remain so, but tend not only to increase from the continued play and action of the heart, but one will produce another, hypertrophy will give rise to valvular disease and inflammation of the endocardium, while the converse is also true, and to a much greater degree. Functional diseases of the heart have in themselves little power in shortening life, but as they are at times causes of the organic affections, the indirect influence is at times very pernicious.

*Influence of age and sex.*—The age has a strong influence in favouring the development of heart disease, while the masculine sex is also not without its influence. Men are much more exposed to the causes of inflammation than women, and therefore suffer more from all the disorders which arise from it; nutrition is also more active in them, favouring, of course, the development of hypertrophy, and of other affections in which nutrition is in excess. As regards the influence of age, it may be readily analysed; cardiac diseases must be proportionably more numerous as we advance in life, 1st, because they are of slow growth, and form, as it were, insensibly, so that they only reach their full development after many years; 2d, because the lesions of nutrition are in themselves more frequent in the old than in the young, as is proved by the invariable and natural increase of the heart as we advance in years, even if no absolute disease be developed. Males are, therefore, more subject to cardiac diseases than females, partly from the greater exposure to the causes of inflammation, and partly from the violent efforts to which the heart is subjected in many of the laborious occupations of the male sex. This, however, holds good only with the organic diseases of the heart, for the nervous functional disorders are vastly more frequent in women, especially in those in whom the nervous susceptible character is most developed.

*General Diagnosis and Prognosis.*—Although accurate or special distinctions as to the precise seat of heart disease and its probable termination, can only be made by studying carefully the physical conditions of this organ, and the precise part affected, there are certain general characters of heart disease which are well known in their applications to the general study of these affections.

Besides the special symptoms of organic diseases of the heart, they are generally known by some decided signs which indicate that some serious mischief has attacked the organs. These are orthopnœa, a feeling of weight and of stricture in the præcordia, fulness of the cervical veins, and great increase of dyspnœa in ascending a height or a steep flight of stairs. Blueness or lividity of the lips is also an excellent sign. A thrilling pulse, and œdematous effusions are also often characteristic. The irregularity of the pulse and the violence of the palpitations are common to both nervous and organic diseases. Pain confined to a limited spot near the apex of the heart is much more common in nervous affections; the same is true of a sensation of fluttering at the heart, of shortness of breath, proportionate merely to the palpitation, and differing from the violent dyspnœa of organic disease. The probability of nervous disorder is rendered much greater if the patient present other signs of a nervous temperament, especially if called into action by the usual exciting causes. The mode of origin of the disease is also important for diagnosis; if at first inflammatory, it is probably organic; or if the patient be stout and muscular, and of a family in which diseases of the heart are hereditary, or the gouty and rheumatic diathesis is very strongly developed. On the other hand, both original constitution and the previous existence of a disease capable of disordering the innervation, render the existence of nervous disease probable. An affection of another organ, especially of the lungs, may act in the same way, and give rise to severe disease, which at times may appear to be organic, but will be quickly dissipated if the original disease be removed: this is very frequently the case with affections of the lungs, especially if the left lung be much indurated, and thus impedes the action of the heart, and conduct the sounds and impulsion both to the ear of an observer and throughout the chest.

The general prognosis of heart disease is commonly understood to be highly unfavourable; hence, in ordinary language, a person who is labouring under an affection of the heart is supposed to be incurably diseased. This is no doubt true, as regards the extreme disorganization of the valves, and of the internal membrane of the heart and aorta, as well as very decided hypertrophy and dilatation; but it is not true of acute inflammatory affections of the heart, or of the moderate degrees of hypertrophy, and still less of the sympathetic nervous disorders which so frequently require medical aid, and often excite the greatest apprehension. Even in those forms of disease in which a strict cure is not expected, the symptoms may, after a time, cease to increase, and even positively decline, without apparently shortening life. Hence the prognosis really depends upon a special diagnosis, and is, in fact, included in it; and if the nature of the disease of the heart is once ascertained, and its rate of increase or diminution settled, the prognosis may be defined—provided no circumstances of a disturbing kind should arise.

